# Improving Animal Monitoring Using Small Unmanned Aircraft Systems (sUAS) and Deep Learning Networks

**DOI:** 10.3390/s21175697

**Published:** 2021-08-24

**Authors:** Meilun Zhou, Jared A. Elmore, Sathishkumar Samiappan, Kristine O. Evans, Morgan B. Pfeiffer, Bradley F. Blackwell, Raymond B. Iglay

**Affiliations:** 1 Geosystems Research Institute, Mississippi State University, Oxford, MS 39762, USA; mz271@msstate.edu; 2 Department of Wildlife, Fisheries and Aquaculture, Mississippi State University, Box 9690, Oxford, MS 39762, USA; kristine.evans@msstate.edu (K.O.E.); ray.iglay@msstate.edu (R.B.I.); 3 U.S. Department of Agriculture, Animal and Plant Health Inspection Service, Wildlife Services, National Wildlife Research Center, Ohio Field Station, Sandusky, OH 44870, USA; morgan.b.pfeiffer@usda.gov (M.B.P.); bradley.f.blackwell@usda.gov (B.F.B.)

**Keywords:** drone, RPA, UAV, UVS, CNN, ResNet, machine learning

## Abstract

In recent years, small unmanned aircraft systems (sUAS) have been used widely to monitor animals because of their customizability, ease of operating, ability to access difficult to navigate places, and potential to minimize disturbance to animals. Automatic identification and classification of animals through images acquired using a sUAS may solve critical problems such as monitoring large areas with high vehicle traffic for animals to prevent collisions, such as animal-aircraft collisions on airports. In this research we demonstrate automated identification of four animal species using deep learning animal classification models trained on sUAS collected images. We used a sUAS mounted with visible spectrum cameras to capture 1288 images of four different animal species: cattle (*Bos taurus*), horses (*Equus caballus*), Canada Geese (*Branta canadensis*), and white-tailed deer (*Odocoileus virginianus*). We chose these animals because they were readily accessible and white-tailed deer and Canada Geese are considered aviation hazards, as well as being easily identifiable within aerial imagery. A four-class classification problem involving these species was developed from the acquired data using deep learning neural networks. We studied the performance of two deep neural network models, convolutional neural networks (CNN) and deep residual networks (ResNet). Results indicate that the ResNet model with 18 layers, ResNet 18, may be an effective algorithm at classifying between animals while using a relatively small number of training samples. The best ResNet architecture produced a 99.18% overall accuracy (OA) in animal identification and a Kappa statistic of 0.98. The highest OA and Kappa produced by CNN were 84.55% and 0.79 respectively. These findings suggest that ResNet is effective at distinguishing among the four species tested and shows promise for classifying larger datasets of more diverse animals.

## 1. Introduction

Animals colliding with aircraft pose significant risks for animal and human safety, as well as serious costs for aviation when strikes occur [1,2]. Here, we define risk in its basic form as the likelihood of a collision with the likelihood of predefined damage or negative effects [3]. Airport biologists and personnel attempt to mitigate these risks by deterring certain species from airports by habitat modification, fencing, translocation, auditory or visual deterrents, and population control, but identifying animal area use and prioritizing management actions can be difficult [4,5]. Animal monitoring is routinely conducted on many airports, but bias varies among human observers, and frequent monitoring is sometimes unattainable due to time and funding constraints and the amount of area needing to be covered [5,6]. We suggest that there is opportunity to couple traditional animal survey methodology (e.g., avian point counts) with novel animal sampling techniques to survey airports with potentially minimal bias and effort [7].

Small unmanned aircraft systems (sUAS) have recently emerged as a potential solution for safely conducting accurate animal surveys among multiple human observers [8,9,10,11,12,13]. They enable users to safely and easily access and cover expansive areas with fine spatial and temporal resolutions while reducing labor costs and user bias [14,15,16,17,18,19,20]. Manual image analysis by humans is one of the primary constraints of sUAS for monitoring animals because sorting and analyzing large amounts of imagery that can be collected in minimal time (e.g., >1000 images) without missing animals takes a large amount of time [21]. Most biologists do not have the time or personnel resources to devote to manually analyzing these images, so image analysis is often not even conducted.

Previous research suggests that automated classification techniques can classify camera trap imagery [22,23], thus, it is reasonable to apply similar processes to imagery obtained via sUAS [23,24]. Indeed, automated machine learning techniques have been used to classify animals quickly and accurately from high resolution sUAS-collected imagery [25,26,27,28]. A previous study comparing unsupervised and supervised classification approaches determined that a supervised learning approach using linear discriminant analysis and a symbolic classifier outperformed unsupervised approaches like principal component analysis and K-means clustering [29]. Supervised deep learning algorithms, such as convolutional neural networks (CNN), outperform traditional supervised machine learning techniques such as support vector machines in learning distinctive features from data [30]. CNNs use a series of convolutional layers to filter the input into higher level features. While base deep learning classification algorithms have produced 60–80% classification accuracy between objects in the past, deeper neural networks with up to 152 layers have been demonstrated recently to perform better at classification tasks [31]. These networks can extract more features and improve upon the classification generated from CNNs, which is especially useful for discerning animals from an aerial viewpoint as there are less features to work with than traditional image classification problems. While deep neural networks require immense training, a technique called residual learning can ease the training cost. Residual learning happens through reformatting the learning layers as learning residual functions with sequential reference to the previous layer inputs, rather than learning unreferenced functions. This allows for deep neural networks to maintain a relatively low complexity and demonstrates higher accuracy than traditional CNNs [32].

The objective of this study is to compare the efficacy of different deep learning frameworks on animal imagery collected using sUAS. Based on the methods found in literature, two deep learning frameworks were compared in order to determine best practices for classifying animals quickly and accurately from sUAS-collected imagery in airport-like environments. We expected the deep learning approach to be able to accurately classify between the four animal species as well as the ResNet algorithm to outperform the traditional CNN approach in terms of classification accuracy.

## 2. Materials and Methods

### 2.1. Study Area and Image Collection

**Figure 1:**
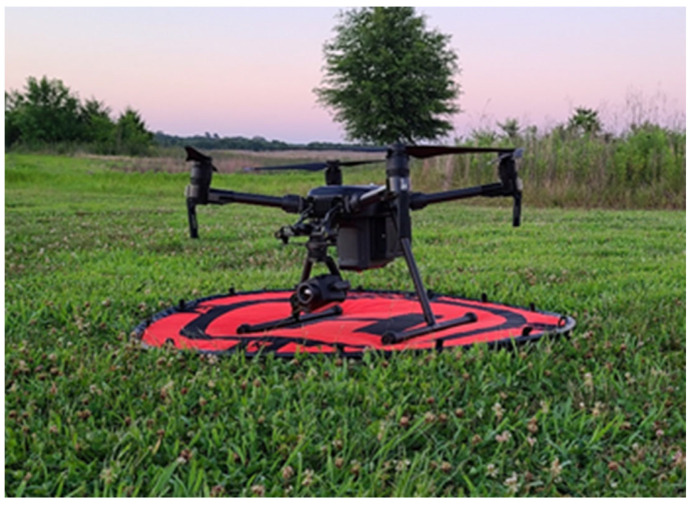
Small, unmanned aircraft system used in this study—Quadcopter—DJI Matrice 200 V2—equipped with visible/thermal sensor payload—Zenmuse XT2—that was used to capture imagery.

We collected images in the visible spectrum (RGB) using either a DJI Zenmuse XT2 with an 8 mm visual lens (640 × 512 25 mm lens thermal camera) or a DJI Zenmuse X7 with a 35 mm lens mounted on a multirotor DJI Matrice 200 V2 (SZ DJI Technology Co., Ltd., Shenzen, China, Figure 1). Flights were conducted using both manual and autonomous flight modes with a DJI Cendence remote controller and the DJI Pilot app on Android software with a Samsung T500 tablet. Autonomous flights were conducted using a lawnmower pattern with 60% overlap, and in both autonomous and manual flight, images were taken at 2 s intervals with the gimbal at nadir (90 degrees or straight down) angle. A lawnmower pattern covers an entire survey region evenly and follows a traditional back-and-forth path of a lawnmower [33]. All other settings were automatically applied through the DJI Pilot app.

**Figure 2:**
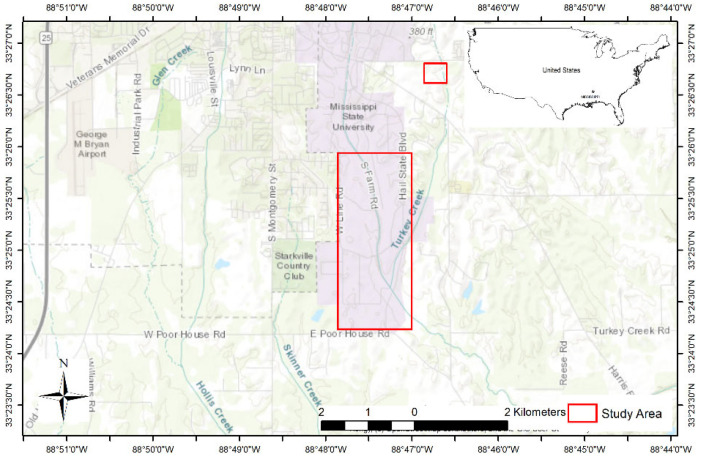
Study area (shown in red wire frame)—Mississippi State University properties—Cattle pastures, row crops, captive facilities, and small farm ponds.

Flights were conducted at varying altitudes of less than 60 m above ground level (AGL), but high enough to avoid disturbing animals, over Mississippi State University properties (33.45626, −88.79421) between January and April 2021 including cattle pastures, row crops, captive facilities, and small farm ponds (Figure 2). The total study area was approximately 6.2 square kilometers. We selected flight altitudes based on previous research [34,35,36] concerning animal disturbance to UAS and operational considerations in an airport environment.

The aforementioned combinations of sensors and flight parameters were chosen to generate high resolution images among the different animals. We selected four groupings of domestic and wild animals for this study, horses (*Equus caballus*), white-tailed deer (*Odocoileus virginianus*), cattle (*Bos taurus*), and Canada Geese (*Branta canadensis*). Our selections offered us accessibility (all species) as well as opportunities to incorporate potential hazards to aircraft (white-tailed deer and Canada Geese) [1]. There was minimal movement among animals during the collection of imagery. All flights were conducted during daylight hours with optimum weather conditions (e.g., partly sunny to sunny, <35 kph average wind speed and gusts, >5 km visibility) and following all U.S. Federal Aviation Administration Part 107 regulations.

### 2.2. Image Processing

On returning from the field, we transferred images from onboard SD cards to an external hard drive for storage and then to a local hard drive for manipulation. Image resolution (cm/pixel) or ground sample distance (GSD) was variable since it depended on AGL and sensor specifications, but all images had GSDs < 1.4 cm/pixel. Briefly, GSD is the distance between the center points of adjacent pixels and a smaller GSD value equals higher resolution. The images do not have the same GSD because they were obtained from differing altitudes and from two different lenses. No image enhancement or other preprocessing was performed on the collected imagery because we wanted to test our algorithms on base imagery captured from a sUAS. Because deep learning models need to be trained on a set of square imagery, we cropped out square images from the collected RGB aerial images as close to individual animals as possible without including shadows using Microsoft Photos (Microsoft Corporation, Redmond, Washington, U.S.). Several aerial images contained more than one animal per image. We cropped 100 images of individual animals per animal class among the four species, resulting in 400 total images of individual animals.

**Figure 3:**
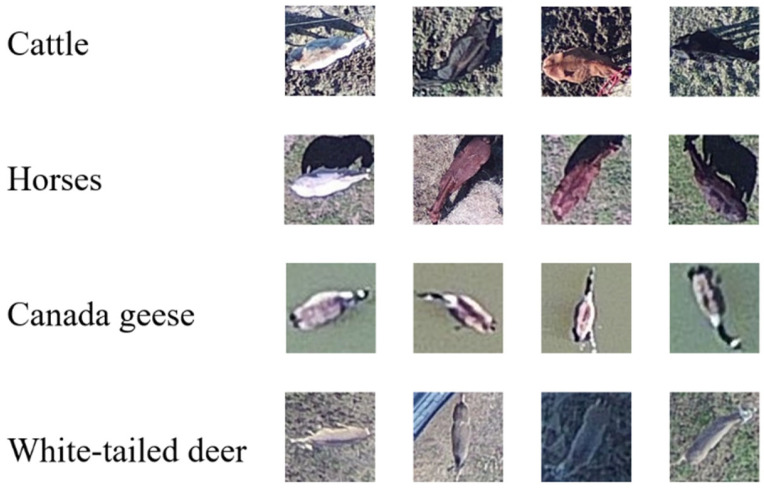
Sample images from dataset used in study.

Despite images sometimes containing the same individual animal, each picture was a unique posture or position (Figure 3). Only full-bodied images of animals were used for our experiments (Figure 3). Our intention in this effort was to demonstrate automated identification, not to move towards fully developed, bias-corrected survey methodology. We then used a cross-validation Jacknife [37] script to separate the cropped imagery folder into training and testing data. The script randomly selected among images using a random number seed to split the whole image set, preventing individual bias that may occur if the training set was manually selected. Training data were used to train the neural network models which were then tested using the testing data to determine the accuracy of the model. All images were then readjusted to the same size before training.

### 2.3. Deep Learning

#### 2.3.1. Convolutional Neural Network

The convolutional neural network (CNN) is a type of deep learning model used for image classification tasks. The CNN transforms an input image into a feature map representation using a cascade of modules each performing three operations, (1) convolution filtering, (2) rectified linear unit (ReLu), and (3) pooling. The convolution operation takes the input raw pixel map or a feature map and applies filters or kernels to compute the convolved feature map. Kernels are functions represented by 3 × 3, 5 × 5 or 7 × 7 matrices composed of different directional filters. These filter sizes were taken from a previously used CNN example used to classify images from a CIFAR-10 dataset [38].

During the training process, CNN learns the optimal values for kernel functions to enable the extraction of useful features from the input map. In each module, CNN could employ or learn more than one filter to efficiently extract the feature maps. The number of filters is directly proportional to number of feature maps the CNN extracts from input, the amount of computational space, and time. After convolutional filtering, CNN applies ReLu to extracted feature maps to introduce nonlinearity into the learning. This is a simple threshold function where ReLu(x) = max (0, x), returns an output of x when the value of x > 0, and an output of 0 when the value of x < 0. The ReLu step is always followed by the pooling step where the CNN down samples the feature map to reduce the size and thereby the computation in next stages. Several pooling methods are mentioned in the literature and max pooling is commonly used [39]. In max pooling, the output map is generated by extracting a maximum value of the feature map from extracted tiles of a specified size and stride. The last step in the CNN is a full connected neural network to learn the feature maps extracted through convolutional filters. Additional details on the architecture of the CNN may be found in Table 1. The CNN configuration we used contains 61,496 training parameters [40].

#### 2.3.2. Deep Residual Learning Networks

Deep learning architectures such as CNNs could perform better by introducing more modules of convolutional filters, ReLu, and pooling into the architecture [31].The performance improvement in training error achieved by adding deeper layers is often eclipsed by poor overall optimization. This degradation in training is not caused by the overfitting of data [31]. In traditional deep learning networks such as CNN, the number of layers of image features is increased through convolutional filtering and the resolution is decreased through pooling. In deep residual neural networks (e.g., ResNet), a deeper model is constructed by adding identity mapping layers, while the other layers are copied from traditional (shallow) deep learning architecture [31]. In this way, the deeper network constructed by using identity layers will not produce a training error that is higher than the error rates of the shallower architecture. Additional details on the architecture of the two ResNet algorithms may be found in Table 2. We chose the two examples of ResNet, ResNet 18 and ResNet 34, which have 18 and 34 layers respectively. These are two popular implementations studied widely for the image classification problem [32,41]. Different layer sizes and number of layers were not tested for this study. Our ResNet configurations contained 11,689,512 and 21,797,672 training parameters for ResNet 18 and ResNet 34 respectively [40].

### 2.4. Image Augmentation

Deep learning classifiers require large amount of training images to achieve good performance. Sometimes, this can be solved by using image augmentation where more training images are artificially created through rotation, and flip. In this work, to improve the number of training samples for CNN, ResNet18 and ResNet34, we employed two augmentation techniques. First is random rotation where the images are rotated between 0 and 180 degrees, and in second technique, half of the training samples are flipped horizontally [42].

### 2.5. Experimental Setup

We used the aforementioned algorithms and varied several parameters to test the effect of learning rates and epoch sizes on classification accuracy. Two training and testing splits were used, 10–90 and 20–80, resulting in 10 and 20 training images paired with 90 and 80 testing images respectively. After testing various training percentages ranging from 5% to 50% (5–50 images) in increments of 5%, we observed that 10% training samples (10 images) provided a fairly high accuracy and chose this as our default training percentage. We also observed that increasing the number of training samples past 20% did not significantly improve the overall accuracies of the algorithms.

Three different learning rates were compared in this set of experiments. The deep learning neural network models used in this study were trained using the stochastic gradient descent (SGD) algorithm [43]. SGD optimizes the current state of the model by estimating the error gradient using the training samples and updating the weights using backpropagation. Learning rate affects how much the model changes in response to the estimated error from the model weights updating in each epoch. Choosing the appropriate learning rate is crucial as too small of a learning rate may result in a longer training process without a significant increase in accuracy. However, a value that is too large may result in unstable training due to converging too fast to a subpar solution leading to lower accuracy [44]. Typical learning rates used in training neural networks are between 0.0 and 1.0 [45]. The learning rate is considered one of the most important parameters of the model and we considered this rate carefully in our approach [46]. Specifically, we tested learning rates of 0.0001, 0.001 and 0.1. Other studies have traditionally studied these learning rates, so we used them for our experiments [47]. A learning rate of 0.0001 was chosen as the starting learning rate because it was the default learning rate for the PyTorch SGD algorithm [48].

The number of epochs sets the number of times that the learning algorithm will traverse through the training set [47]. Epochs are typically set as large numbers for the algorithm to run until the model is sufficiently optimized [47]. Typically, higher accuracies are expected as the epoch sizes increase, but there is a limit where the network begins to become over-trained and would not benefit from more training epochs [47]. We empirically determined our epoch experiments by testing a range of epochs from 5 to 100 in increments of 10 epochs for the ResNet algorithms. The ResNet algorithms produced a fairly high accuracy around the 25-epoch mark but did not display much improvement after increasing past 100 epochs. The CNN epoch size of 1000 was determined by adjusting until the run time was comparable to the ResNet model run times.

We compared our algorithms and the various adjusted parameters using both overall accuracy (OA) and Kappa statistic. Overall accuracy helps us understand how many pictures were misclassified and Kappa statistic gives us a measure of how different the observed agreement is from the expected agreement [49]. All experiments were performed on a 64-bit Intel^®^ Core™ i7-8550U Windows CPU with 16 GB of RAM.

## 3. Results

### 3.1. Collected Imagery

We conducted seven different flights and collected 3438 total images of which 1288 contained one or more animals. We captured 183 images of horses (range 1–15 individuals per image), 61 images of white-tailed deer (range 1–2 individuals per image), 939 images of cattle (1–20 individuals per image), and 105 images of Canada Geese (1–12 individuals per image). Of these aerial images collected, numerous images contained more than one animal. We only chose 100 animals from these aerial images for the purpose of these experiments.

### 3.2. Deep Learning Algorithm Comparisons

No consistent learning rate was found that provided the best accuracy for the CNN algorithm. For the 10% training, a learning rate of 0.01 produced the highest accuracy, while for the 20% training, a learning rate of 0.001 produced the highest accuracy (Table 3). However, the learning rate of 0.001 for both ResNet algorithms consistently provided the highest accuracy for both training splits compared to other learning rates of 0.0001 and 0.01 (Table 3).

In this set of experiments comparing the effect of varying the epoch size (Table 4), the default learning rate of 0.0001 studied above was used as the baseline learning rate. The CNN run on 10% training sample had the best accuracy of 71.27% when run using the highest epochs of 1000. The best accuracy for the CNN trained on 20% of samples had the highest accuracy of 75.53% when run for 150 epochs. For both ResNet algorithms trained on 20% of the samples, training for the largest number of epochs resulted in the best accuracy. However, for the 10% training samples, ResNet 34 converged at 100 epochs and did not benefit from further training. All algorithms were run at the default learning rate of 0.0001.

In this set of experiments comparing the effects of varying the epoch size (Table 5), the experiment shown in Table 4 being repeated with random rotation image augmentation. The CNN with 10% training data showed an improvement of approximately 1.3% of OA and 0.04 of Kappa whereas with 20% training data, random rotation image augmentation improved OA by almost 10% and Kappa by 0.14 which is significant. With ResNet classifiers, the improvement in overall accuracy is not significant. Both ResNet algorithms trained on 10 and 20% benefitted from training with the most epochs, peaking at 99.18% for ResNet 18 and 98.91% for ResNet 34.

In this set of experiments comparing the effects of varying the epoch size (Table 6), the experiment shown in Table 4 being repeated with horizontal flip image augmentation. The improvement offered by horizontal flip augmentation clearly significant with the CNN than ResNet. The CNN produced OAs of 72.64% and 84.55% for 10 and 20%. The ResNet 18 algorithm produced accuracies of 97.56% and 99.18% for 10 and 20% training percentages, respectively. The ResNet 34 algorithm produced accuracies of 97.56 and 98.91% for the 10 and 20% training percentages.

## 4. Discussion

Our results demonstrated that all three deep learning algorithms can accurately classify four animal species captured from aerial imagery. Upon further comparison between CNN and ResNet algorithms, ResNet consistently produced better OA and Kappa compared to the plain CNN. ResNet 18 was able to train faster than the ResNet 34 due to the smaller number of layers. Despite the faster training time and a smaller number of layers, ResNet 18 still managed to remain comparable or favorable to ResNet 34 for this classification problem. The larger number of neural network layers in ResNet algorithms likely provide a more robust classification of species. However, ResNet 34 may be too complex when training samples are scarce. ResNet with 34 layers did not converge as well as ResNet with 18 layers when trained with 10% of samples. A base CNN is also not ideal for this problem due to its need for many training samples [50].

From our learning rate experiments, we gathered that finding an optimized learning rate is crucial for the ResNet algorithms. ResNet 18, increasing the learning rate by a factor of 10 from 0.0001 to 0.001, improved accuracy by 2.7%. This is relatively insignificant when compared to the 23.85% decrease in accuracy when the learning rate is further increased by a factor of 10 to 0.01 for ResNet 18. Having a 0.01 learning rate, which changes the weights 100× the rate of 0.0001, led both ResNet networks to misclassify more animals. Due to the limited number of training samples used, smaller learning rates are favorable for the ResNet algorithm rather than larger, which is supported by previous research [51]. The CNN network did not converge as much with smaller learning rates, but the largest learning rate caused the network to overshoot the weights and reduce accuracy. Other studies have found similar trends where smaller learning rates memorizes easy-to-generalize patterns well and outperform larger learning rates [52]. While this classification problem was relatively easy due to the many visible distinctions among the four animal species studied, future studies involving classification of subtly different species, such as Great Egrets (*Ardea alba*) compared to Snowy Egrets (*Egretta thula*), may prove difficult for the CNN with smaller learning rates due to the lack of generalization in patterns.

Increasing training data improved the overall accuracies of most of our algorithms drastically, by upwards of 5%. Several other studies have demonstrated the need for larger training samples [53,54,55], with some suggesting data augmentation to solve data deficiency issue [53,54]. We chose to test the performance of our models on a low number of training samples along with two different augmentation techniques in order to determine the efficacy of the algorithms. Despite the relatively low number of training samples, our algorithms were able to produce fairly high accuracies, ranging from 71% to 98% between the CNN and ResNet algorithms. With the use of random rotation and horizontal flip augmentation the accuracies were improved significantly (Table 5 and Table 6).

For the CNN algorithm, increasing the epochs resulted in higher accuracy by allowing the model to learn the general pattern [56]. A drastic improvement in accuracy was seen for the CNN algorithm trained on 20% samples with only a 50 epochs increase from 100 to 150, but accuracy decreased when epochs increased further past 150. This is likely due to the increased number of training samples providing additional valuable information to the network, allowing the model to converge much earlier. However, the model becomes overfit when the epochs are increased past 150, which occurs when the network is too optimized on the training data and misses a more general trend [57]. For the ResNet algorithms, increasing the epoch sizes did not result in significant accuracy improvements, (<5%). The high classification accuracy demonstrated by the ResNet algorithm indicates that the model is fairly well optimized to the classification problem within 25 epochs.

**Figure 4:**
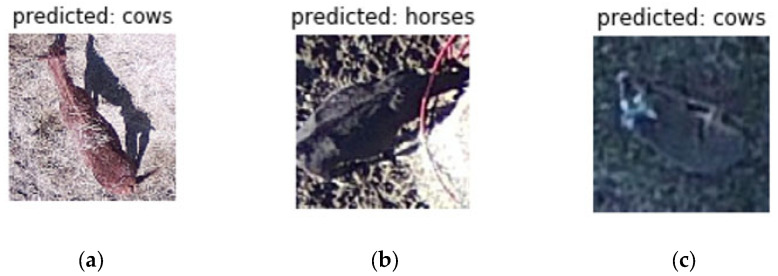
Examples of misclassified images of (**a**) a horse, (**b**) cattle, and (**c**) a white-tailed deer.

The majority of misclassifications produced by algorithms were between animals with similar body types. Notably cows, horses, and deer occasionally show similar body structure (Figure 4). The misclassifications may be most alleviated by increasing the number of training samples. While learning rates and epoch sizes are important, in our case, the amount of training samples consistently led to improved accuracies. In addition to more training samples, approaches involving thermal imagery or pre-filtering images to improve feature extraction before feeding into the network may also decrease misclassifications [53,54,58]. Another consideration for misclassifications involves sensitivity to body positions as well as animal movements. Our deep learning algorithms are both rotation and scale invariant as they were trained on images with a variety of different body rotations and postures. Bias due to movement of animals was also not considered due to the still imagery being captured with high shutter speeds. Despite our best efforts to remove shadows from imagery, shadows still remained a factor in some of the misclassifications. As shown above in Figure 3a, the black shadow of the horse may have caused the network to classify the overall image as a black cow. In addition, the background of the photo may have also impacted the accuracy of these classifications. For our experiments, this was unavoidable as the network models required square images.

The algorithms tested in these experiments took minimal time to run, with 2 h being the longest run time. The number of training parameters for the CNN is significantly smaller than either ResNet algorithms, around 60,000 compared to 11 and 21 million parameters for ResNet 18 and 34 respectively. This drastic difference in the number of training parameters led to a large difference in run time between the two types of algorithms. Increasing the sample sizes would also increase the run time as the model needs more time to train on a higher number of samples.

Our results comparing algorithms, learning rates, and epoch sizes demonstrates the utility of CNN and ResNet algorithms for animal classification and sets a foundation for future studies to classify among different animals. As evidenced in all the experiments, having more training samples leads to higher classification accuracies. As researchers collect more imagery using sUAS and build aerial imagery repositories, neural network algorithms will benefit from having a more robust set of images to provide accurate weight adjustments to the model [53,54,55]. This high level of accuracy compliments traditional wildlife surveys by accurately classifying animal species and has the potential to assist in estimating relative abundance in airport land covers [7]. Automated classification will then aid wildlife managers and airport personnel by decreasing the workload and time required to sort through large amounts of sUAS collected imagery, contributing data to strike risk assessments [6], and better informing prioritization of animal management actions to reduce animal strikes with aircraft [8,9,10,11,12,13].

## 5. Conclusions

Our study demonstrates that visible imagery collected at 60 m or less is adequate for accurately classifying four animal species. We used two readily accessible species and two species ranked as airport hazards. We demonstrated that CNN and ResNet both offer high classification accuracies even with small amounts of training samples. Increasing training sample sizes improves the networks, but training sizes between 10 to 20 images per class are adequate for learning animals in our study from an aerial perspective. Future studies using larger datasets with more species along with more deep learning algorithms will improve automated classification of animals from aerial imagery.

## Figures and Tables

**Table 1:** The architecture of the convolutional neural network (CNN) used for classification of 400 sUAS images of cattle, horses, white-tailed deer, and Canada Geese, a subsample of the 1288 images collected that contained animals.

Layer	Layer Name	Output Size	Layer Info	Processing
1	2D Convolution	28 × 28	5 × 5, 6, stride 1	Input 32 × 32, ReLu
2	Pooling	14 × 14	2 × 2 Max Pooling, stride 2	
3	2D Convolution	10 × 10	5 × 5, 16, stride 1	ReLu, stride 1
4	Pooling	5 × 5	2 × 2 Max Pooling, stride 2	2 × 2 Max Pool, stride 1
5	Fully Connected ANN	4 × 1	Cross Entropy Loss, 0.9 Momentum	ReLu

**Table 2:** The architectures of the deep residual neural networks (ResNet 18 and ResNet 34) used for classification of on 400 sUAS images collected of cattle, horses, white-tailed deer, and Canada Geese, a subsample of the 1288 images collected that contained animals.

Layer	Layer Name	Output Size	ResNet 18	ResNet 34	Processing
**1**	2D Convolution	112 × 112	7 × 7, 64, stride 2 3 × 3 Max Pooling, stride 2		Input 224 × 224, ReLu
**2**	Pooling	56 × 56			
**3**	2D Convolution	56 × 56	$\left[\begin{array}{cc} 3\times 3 & 64\\ 3\times 3 & 64 \end{array}\right]\times 2$	$\left[\begin{array}{cc} 3\times 3 & 64\\ 3\times 3 & 64 \end{array}\right]\times 3$	ReLu
**4**	2D Convolution	28 × 28	$\left[\begin{array}{cc} 3\times 3 & 128\\ 3\times 3 & 128 \end{array}\right]\times 2$	$\left[\begin{array}{cc} 3\times 3 & 128\\ 3\times 3 & 128 \end{array}\right]\times 4$	ReLu
**5**	2D Convolution	14 × 14	$\left[\begin{array}{cc} 3\times 3 & 256\\ 3\times 3 & 256 \end{array}\right]\times 2$	$\left[\begin{array}{cc} 3\times 3 & 256\\ 3\times 3 & 256 \end{array}\right]\times 6$	
**6**	2D Convolution	7 × 7	$\left[\begin{array}{cc} 3\times 3 & 512\\ 3\times 3 & 512 \end{array}\right]\times 2$	$\left[\begin{array}{cc} 3\times 3 & 512\\ 3\times 3 & 512 \end{array}\right]\times 3$	
**7**	Fully Connected ANN	1 × 1	Cross Entropy Loss, 0.9 Momentum		Average Pool, Softmax

**Table 3:** Comparison of overall accuracy, Kappa statistic, and run time for CNN, ResNet 18, and ResNet 34 with three different learning rates and two different training sizes.

		10% Training Samples			20% Training Samples		
Algorithm	Learning Rate	Run Time	OA	Kappa	Run Time	OA	Kappa
CNN (1000 epochs)	0.0001	13 m 30 s	71.27%	0.59	19 m 38 s	74.38%	0.65
	0.001	9 m 21 s	66.12%	0.53	9 m 44 s	**80.54%**	0.68
	0.01	9 m 24 s	**72.62%**	0.63	9 m 59 s	78.72%	0.66
ResNet 18 (25 epochs)	0.0001	9 m 8 s	94.04%	0.92	9 m 47 s	94.59%	0.93
	0.001	10 m 8 s	**96.74%**	0.96	9 m 58 s	**97.89%**	0.97
	0.01	8 m 14 s	72.89%	0.63	9 m 42 s	85.71%	0.80
ResNet 34 (25 epochs)	0.0001	17 m 17 s	93.04%	0.90	17 m 32 s	96.06%	0.96
	0.001	15 m 26 s	**97.83%**	0.97	16 m 53 s	**98.48%**	0.98
	0.01	14 m 15 s	68.56%	0.54	17 m 11 s	59.89%	0.45

Best accuracies are bolded.

**Table 4:** Comparison of overall accuracy, Kappa statistic, and run time for CNN, ResNet 18, and ResNet 34 with four different epoch sizes, two different training sizes, and no augmentation.

		10% Training Samples			20% Training Samples		
Algorithm	Epochs	Run Time	OA	Kappa	Run Time	OA	Kappa
CNN (0.0001 LR)	100	1 m 30 s	60.07%	0.50	1 m 30 s	65.54%	0.51
	150	2 m 11 s	58.98%	0.49	3 m	**75.53%**	0.66
	200	2 m 50 s	66.67%	0.53	3 m 45 s	72.64%	0.60
	1000	13 m 30 s	**71.27%**	0.59	19 m 38 s	74.38%	0.65
ResNet 18 (0.0001 LR)	25	9 m 8 s	94.04%	0.90	9 m 47 s	94.59%	0.93
	50	36 m 39 s	94.03%	0.90	43 m 42 s	98.48%	0.98
	100	73 m 23 s	95.93%	0.93	83 m 18 s	98.17%	0.97
	200	147 m 8 s	**96.20%**	0.94	158 m 16 s	**98.78%**	0.98
ResNet 34 (0.0001 LR)	25	17 m 17 s	93.04%	0.92	17 m 32 s	96.09%	0.95
	50	41 m 20 s	97.87%	0.97	42 m 30 s	96.96%	0.95
	100	83 m 14 s	**98.48%**	0.98	81 m 45 s	97.26%	0.97
	200	166 m 42 s	95.12%	0.92	167 m 11 s	**98.92%**	0.98

Best accuracies are bolded.

**Table 5:** Comparison of overall accuracy, Kappa statistic, and run time for CNN, ResNet 18, and ResNet 34 with four different epoch sizes, two different training sizes with random rotation image augmentation.

Random Rotation		10% Training Samples			20% Training Samples		
Algorithm	Epochs	Run Time	OA	Kappa	Run Time	OA	Kappa
CNN (0.0001 LR)	100	1 m 0 s	44.98%	0.26	1 m 8 s	83.19%	0.77
	150	1 m 29 s	58.26%	0.44	1 m 53 s	81.02%	0.73
	200	1 m 58 s	67.47%	0.56	2 m 13 s	83.19%	0.77
	1000	9 m 53 s	**72.64%**	0.63	11 m 40 s	**84.55%**	0.79
ResNet 18 (0.0001 LR)	25	11 m 27 s	93.22%	0.91	16 m 2 s	96.20%	0.94
	50	21 m 11 s	94.85%	0.93	26 m 11 s	97.83%	0.97
	100	42 m 48 s	96.74%	0.95	73 m 13 s	**99.18%**	0.98
	200	84 m 12 s	**97.56%**	0.96	149 m 16 s	**99.18%**	0.98
ResNet 34 (0.0001 LR)	25	19 m 17 s	89.43%	0.85	30 m 59 s	96.47%	0.95
	50	37 m 10 s	95.66%	0.94	51 m 50 s	98.64%	0.98
	100	74 m 32 s	96.47%	0.95	100 m 42 s	97.56%	0.96
	200	146 m 24 s	**97.56%**	0.96	182 m 50 s	**98.91%**	0.98

Best accuracies are bolded.

**Table 6:** Comparison of overall accuracy, Kappa statistic, and run time for CNN, ResNet 18, and ResNet 34 with four different epoch sizes, two different training sizes with horizontal flip image augmentation.

Horizontal Flip		10% Training Samples			20% Training Samples		
Algorithm	Epochs	Run Time	OA	Kappa	Run Time	OA	Kappa
CNN (0.0001 LR)	100	1 m 2 s	68.83%	0.58	1 m 15 s	78.31%	0.71
	150	1 m 37 s	**71.00%**	0.61	2 m 4 s	84.01%	0.78
	200	2 m 14 s	72.35%	0.63	2 m 31 s	83.19%	0.77
	1000	11 m 0 s	71.54%	0.62	12 m 37 s	81.57%	0.75
ResNet 18 (0.0001 LR)	25	11 m 43 s	93.49%	0.91	14 m 4 s	97.83%	0.97
	50	21 m 29 s	92.41%	0.89	28 m 15 s	**99.18%**	0.98
	100	44 m 1 s	**97.56%**	0.96	61 m 17 s	98.64%	0.98
	200	85 m 42 s	95.66%	0.94	118 m 41 s	98.91%	0.98
ResNet 34 (0.0001 LR)	25	21 m 35 s	96.74%	0.95	23 m 21 s	98.10%	0.97
	50	41 m 33 s	97.01%	0.96	47 m 33 s	98.64%	0.98
	100	80 m 16 s	96.20%	0.94	99 m 55 s	98.64%	0.98
	200	155 m 31 s	97.01%	0.96	195 m 39 s	**99.18%**	0.98

Best accuracies are bolded.

## Data Availability

The data presented in this study are available on request from the corresponding author.
